# Learning the meaning of new stimuli increases the cross-correlated activity of prefrontal neurons

**DOI:** 10.1038/s41598-018-29862-0

**Published:** 2018-08-03

**Authors:** Simon Nougaret, Aldo Genovesio

**Affiliations:** Department of Physiology and Pharmacology, Sapienza University of Rome, Piazzale Aldo Moro 5, 00185 Rome, Italy

## Abstract

The prefrontal cortex (PF) has a key role in learning rules and generating associations between stimuli and responses also called conditional motor learning. Previous studies in PF have examined conditional motor learning at the single cell level but not the correlation of discharges between neurons at the ensemble level. In the present study, we recorded from two rhesus monkeys in the dorsolateral and the mediolateral parts of the prefrontal cortex to address the role of correlated firing of simultaneously recorded pairs during conditional motor learning. We trained two rhesus monkeys to associate three stimuli with three response targets, such that each stimulus was mapped to only one response. We recorded the neuronal activity of the same neuron pairs during learning of new associations and with already learned associations. In these tasks after a period of fixation, a visual instruction stimulus appeared centrally and three potential response targets appeared in three positions: right, left, and up from center. We found a higher number of neuron pairs significantly correlated and higher cross-correlation coefficients during stimulus presentation in the new than in the familiar mapping task. These results demonstrate that learning affects the PF neural correlation structure.

## Introduction

Local and functional populations of neurons, called “cell assemblies”, are considered to represent a diffuse structure delivering facilitation to other system and enhancing the action process^1^. Assemblies dynamically change sizes and connections to encode several types of information^2–4^ as it has been shown in various brain regions^5–10^. In this context, the synchronized firing rate among cells, a reflection of cell-assembly coding, should take part in the learning processes and the assignment of the meaning of an instruction stimulus (IS).

The prefrontal cortex (PF) is important in goal selection, maintenance and monitoring^11–15^ and its role in learning has been highlighted by several neurophysiological^16–22^, lesion and neuropsychological studies^23–28^. The role of learning in PF has been related also to the effect of dopamine. In fact, blocking prefrontal D1^29^ or D2 receptors^30^ affects visuomotor learning but has no effect on familiar associations. Human imaging studies suggest that the PF becomes less engaged as tasks become more automatic^31,32^. Likewise, computational studies^15,33^ implicated the PF in the reinforcement and transfer learning models in which the PF plays has a key role when new rules are learned to replace previous ones^34^.

The present study investigates spike-count correlation associated with learning in two mapping tasks, a new (NovelMap) and a familiar (FamMap) mapping task from data gathered during an earlier study^19^. In these two tasks, three ISs (instruction stimuli) are mapped to three different response targets. In the NovelMap task the monkeys were required to learn the associations or mappings between ISs and response targets during the neural recording while in the FamMap task the recording took place after the mappings were already highly familiar. We studied the correlated activity of the same neuron pairs recorded simultaneously and in both tasks. For this purpose, we computed the joint perievent time histograms (JPETHs)^35–38^ which are two-dimensional histograms displaying the correlated trial-by-trial activity of two simultaneous recorded neurons aligning the activities on different behavioral events. The coincidence histogram, the main diagonal of a JPETH, offers the opportunity to evaluate the level of co-activation of both neurons and the behavioral event related to this coactivation. The ensemble of neurons moves through different levels of coherence and different states as the cognitive requirement of the task change^39^. Consequently, the study of these transient correlations during learning in comparison to well learned associations is important to address whether learning involve broad synchrony among neurons in the PF. Indeed, these transient correlations could facilitate information transfer from one cortical area to another^40^. Such correlations could result from a dynamic re-organization of functional cell-assemblies and arise from changes in the pattern of activity of a large number of neurons^5^ via recurrent excitatory loops within local neural networks^41–43^ that maintain such persistent activity.

## Results

### Behavior

**Figure 1:**
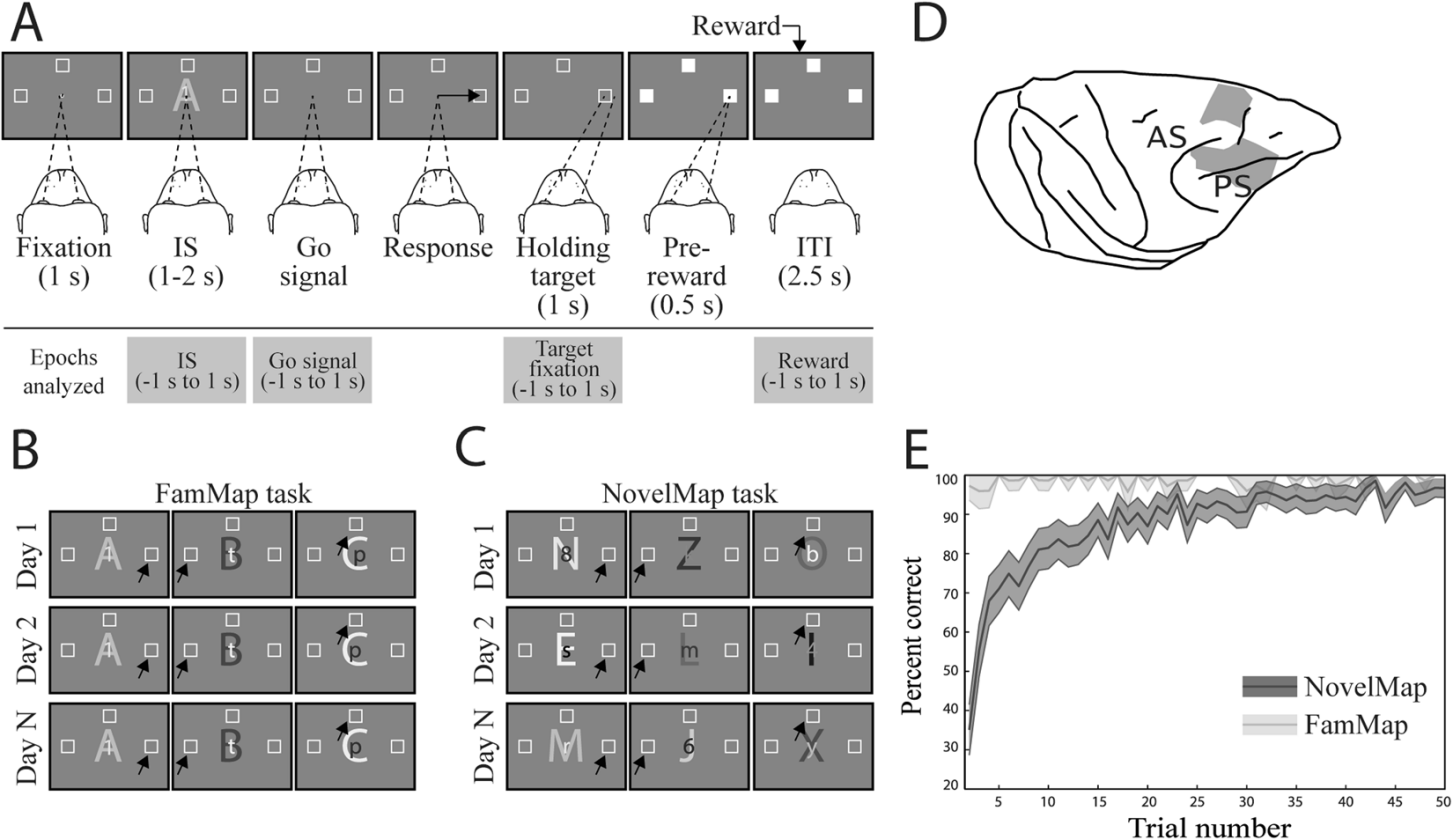
Task design, localization of neuronal recordings, and behavior. (**A**) Upper: Sequence of task events. Gray rectangles represent the computer screen, white dot the fixation spot, white squares the response target locations (not to scale) and “A” represents the instruction stimulus (2 ASCII superimposed characters were presented). The dashed lines represent the gaze angle. The go signal corresponded to the disappearance of the instruction stimulus (1 s, 1.5 s or 2 s after its appearance) and indicated to the monkeys to report their decision with a saccade movement toward the chosen response target (solid arrow). After 1 s of response target fixation and 0.5 s of pre-reward period, the monkey received a liquid reward when appropriate. Lower: Epochs analyzed. From 1 s before to 1 s after, the occurrence of the instruction stimulus, the go signal, the beginning of the fixation of the response target and the reward occurrence. Abbreviations: IS, instruction stimulus; ITI, intertrial interval. (**B**) Examples of a set of stimuli presented during three recording days in the FamMap task. The sets of stimuli were the same across days and the mapping was already well known by the monkeys. (**C**) Same representation as in B but for the NovelMap task. The sets of three stimuli were different across days and the mapping between each stimulus and its corresponding response target had to be learned each day. (**D**) Recording sites. Explored regions are shown in gray. Abbreviations: AS, arcuate sulcus; PS, principal sulcus. (**E**) Behavioral results. Percentage of correct responses in the first 50 trials for each task averaged across the 2 monkeys. The dark gray curve indicates the performance during the FamMap task and the light gray ones the performances in the NovelMap task. Background shading indicates 95% confidence limits.

Two monkeys performed two behavioral tasks, the mapping (NovelMap) and the familiar mapping (FamMap) task (see Materials and Methods and Fig. 1A–C for detailed description). After the monkeys fixed a central fixation spot, three saccade targets appeared in three different positions on the screen. After one second of fixation, a visual instruction stimulus (IS) appeared for 1.0, 1.5 or 2 s and its disappearance served as a “go signal” to perform a saccade to one of the three targets. Monkeys were rewarded only if their response followed the mapping rule for the IS presented. In the FamMap task monkeys encountered only highly familiar ISs and already knew the mapping rule, otherwise they had to learn by trial and error three novel mappings in the NovelMap task.

From the original data of Genovesio *et al*.^19^, the monkey’s behavior was analyzed on sessions with at least 50 completed trials which corresponded to 78 sessions in the FamMap task and to 229 in the NovelMap task. In the FamMap task, both monkeys performed the task accurately (98.6% of correct choices for Monkey 1, 58 sessions; 98.9% of correct choices for Monkey 2, 20 sessions). Considering all the trials together, the monkeys performed the NovelMap task with a high degree of accuracy (92.8% of correct choices for Monkey 1, 126 sessions; 90.8 for Monkey 2, 103 sessions). They learned the novel mappings quickly (Fig. 1E, light gray curve) and on average reached 80% of correct choices after about 10 trials (9^th^ trial for Monkey 1; 10^th^ trial for Monkey 2). Supplementary Fig. 1 shows the behavior of each monkey separately. The behavioral differences between both tasks observed in both monkeys indicates that ISs-response targets associations were already learned in the FamMap task in contrast to the NovelMap task that required learning.

### Database for cross-correlation analysis

From the 307 analyzed behavioral sessions, we selected 92 sessions (46 in each task) including 224 neurons recorded from two monkeys based on the following criterion. We excluded the neuron pairs in which both neurons were recorded from the same electrode to avoid the possibility of contamination. We also excluded neuron pairs recorded less than 50 trials in one of the two tasks. Finally, we excluded neuron pairs with a neuron with a null firing rate during the fixation period in at least one task. The remaining neuronal sample consisted of 439 neuron pairs, 335 from monkey 1 and 104 from monkey 2. During the recording sessions, the action potentials from single units were isolated with quartz-insulated platinium-iridium electrodes (80 µm outer diameter; impedance, 0.5–1.5 MΩ at 1 KHz) driven by 16 electrode microdrive (Thomas Recording, Giessen, Germany) with 518 µm electrode spacing. The signal from each electrode was discriminated by a Multichannel Acquisition Processor (Plexon, Dallas, TX) or offline. The Off Line Sorter (Plexon) was used to scrutinize every unit’s isolation. Individual spike waveforms were accepted only if they clustered clearly in the 3D principal-component space, lacked interspike interval inferior to 1 ms, had waveforms grouped tightly stably in the time domain and clearly differentiated over the course of the recordings.

### Single-pair analysis

**Figure 2:**
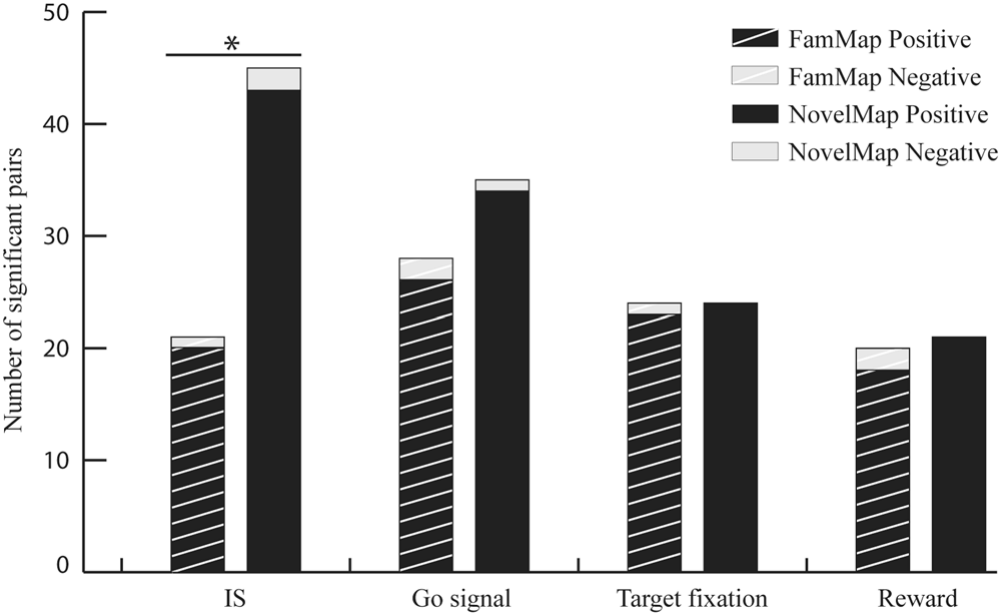
Number of significant correlated pairs of neurons during the 4 task epochs analyzed. Task epochs are from −1 s to 1 s from the occurrence of the IS, the go signal, the beginning of the response target fixation and the reward/no reward occurrence. For each period the left striped bar represents the number of significant correlated neuron pairs during the FamMap task and the right solid bar the number of significant correlated neuron pairs during the NovelMap task. The dark gray part of each bar represents the significantly positive correlated neuron pairs and the light gray part the significantly negative ones (*χ*^2^ test, ^*^p < 0.05).

The analysis of the main diagonal of the normalized corrected covariogram reveals that most of the significant pairs showed a positive correlation and very few a negative one (Fig. 2). Therefore, our analyses focused mainly on the positive correlations. Among the 439 studied pairs, 72 (16%) showed a significant positive correlation during at least one of the four studied epochs when they were recorded in the NovelMap task in contrast to only 49 (11%) when the same neurons were recorded in the FamMap task (χ^2^ test, p < 0.05, χ^2^ = 4.6). Among these pairs, 5% (22/439) showed a positive significant correlation in both cases, representing 31% of the positive significant pairs recorded in the NovelMap task and 45% of those that were significant when recording the FamMap task. The distribution of the number of cross-correlated pairs during both NovelMap and FamMap tasks (Fig. 2) shows a higher number of positively correlated pairs across all the four task epochs during the NovelMap task. However, the number of pairs with positive significant correlation in the NovelMap task was significantly higher than in the FamMap task (χ^2^ test, 43/439 vs 20/439, χ^2^ = 8.28, p < 0.01) only for the IS epoch, whereas the difference was not significant during the other epochs (χ^2^ test, χ^2^ < 0.88, p > 0.05).

**Figure 3:**
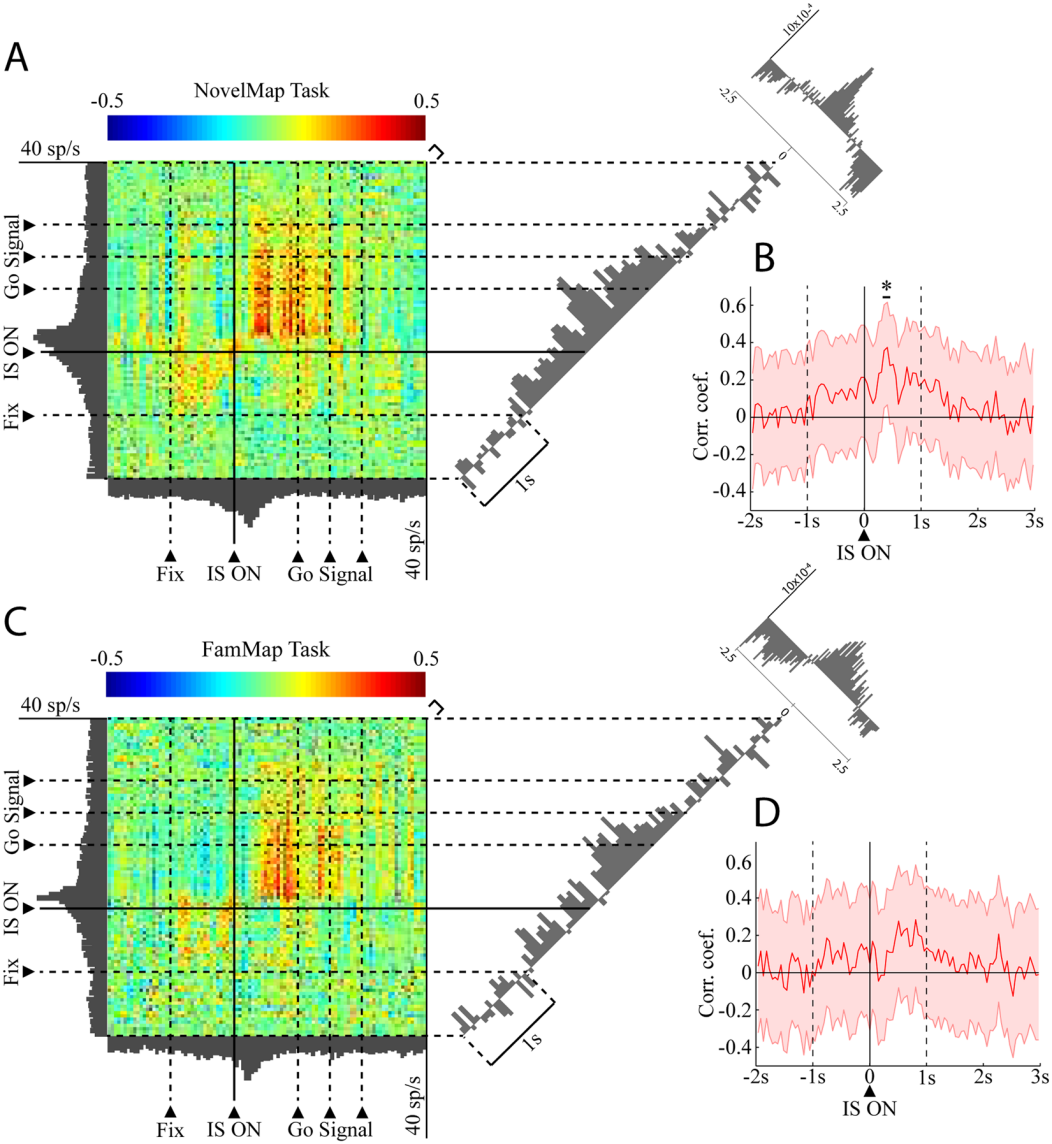
Dynamic modulation of correlated firing in a pair of neurons in both tasks. (**A**) JPETH constructed from 102 trials performed during the NovelMap task. The abscissa and ordinates show the peri-stimulus time histograms for 2 simultaneously recorded neurons. Each pixel of the color-based matrix exhibits the normalized correlation coefficient at a precise time and lag delay relative to the IS onset from blue (negative) to red (positive). The coincidence histogram is plotted to the right of the JPETH. It represents the correlation coefficients along the main diagonal (see the small bracket on the upper right corner of the diagram). The corrected cross-correlogram is presented on the upper right corner of the figure (bin width, 50 ms). (**B**) The coincidence histogram, same representation as in A with the 95% confidence interval corrected by the number of bins for multiple comparison (*, value above the inferior corrected 95% interval). (**C**,**D**) Same neural pair as in A, B recorded in the FamMap Task for 80 trials.

In the IS epoch, 8 neuron pairs exhibited a positive significant correlation during the performance of both tasks, while the 35 remaining pairs with a significant correlation during the NovelMap task were not significant in the FamMap task. Figure 3 shows an example of a pair of neurons significantly correlated in the IS epoch in the NovelMap task (Fig. 3A) but not in the FamMap task (Fig. 3C). For each plot, the activity of the two individual cells is shown as PETH along the abscissa and ordinate axis aligned on the IS onset and the color map indicates the correlated activity. On the right part of the plot, the coincidence histogram is shown for the main diagonal in the interval illustrated by the brackets on the up-right part of the color map. The corrected cross-correlogram is perpendicular to this diagonal. The corrected cross-correlogram is the mean of 1000 “shuffled cross-correlogram” obtained by permutations of the trials, subtracted from the raw cross-correlogram. Both coincidence histograms revealed that there is an increase of the correlated firing of these neurons after the IS onset. However, this increase was significant only during learning in the NovelMap task (Fig. 3B), but not in the FamMap task (Fig. 3D). The cross-correlogram of the same pair of neurons, illustrated in Fig. 3A,C on the top right part of the figure, shows a higher, narrower and more centered on zero peak in the NovelMap task. The cross-correlogram of the same pair using a shorter window of 100 ms of maximal lag and 1 ms bins (Supplementary Fig. 2F,L) shows the existence of a small peak centered on 0 only in the NovelMap task. It provides additional evidence of stronger synchrony in the IS epoch, when the monkey had to map the ISs with the response targets generating and reinforcing the association. Because the normalization that we performed does not entirely rule out the possibility of an influence of the firing rate^44^ we controlled for the firing rate of neurons that make up the pairs. We compared the firing rate of neurons between tasks, during the fixation period and the first second of the presentation of the IS, both when they were part of correlated or uncorrelated pair of neurons (Supplementary Fig. 3). We did not observe any significant difference in the firing rate of the neurons between tasks both during the fixation period (two-sample t-test, t = 0.21, p = 0.83) and during the first second of IS occurrence (two-sample t-test, t = 0.43, p = 0.66) ruling out that differences in firing rate could account for our results. In total, 34 neurons made up the 20 positively correlated pairs of the FamMap task and 65 neurons made up the 43 positively correlated pairs in the NovelMap task. All the neurons that were part of the positively correlated pairs in the FamMap task (34/34) were also included in the uncorrelated pairs and most of the cells part of the positively correlated pairs in the NovelMap task (61/65) were part of the uncorrelated pairs. We compared the firing rate of the neurons that made up correlated and uncorrelated pairs during the first second after IS onset both in the FamMap task and the NovelMap task and did not observe any significant difference (FamMap task: two-sample t-test, t = −0.58, p = 0.56; NovelMap task: two-sample t-test, t = −1.24, p = 0.22).

In addition, for the IS epoch, we evaluated whether the number of significantly correlated pairs was higher than chance level for both NovelMap and FamMap. We randomly selected pairs to create 5 control populations of 439 neuron pairs each. Thus, we generated 5 sets of 439 “constructed” pairs to repeat the test five times for the FamMap task and five times for the NovelMap task. For the FamMap task, we found an average of 7.4 pairs showing a positive significant correlation (10, 10, 5, 6 and 6 pairs) among the 5 random sets. For the NovelMap task, an average of 6.2 pairs (8, 3, 10, 7 and 3 pairs) showed positive significant correlation. The proportion of neurons showing a positive significant correlation was significantly higher than that found by chance not only in the NovelMap task (43/439, 1-sample χ^2^ test, χ^2^ = 215.6, p < 0.001) but also in the FamMap task (20/439, 1-sample χ^2^ test, χ^2^ = 20.124, p < 0.001). There was no significant difference among the number of pairs found by chance between both tasks (paired t test, t = 0.60, p > 0.05).

### Population Analysis

**Figure 4:**
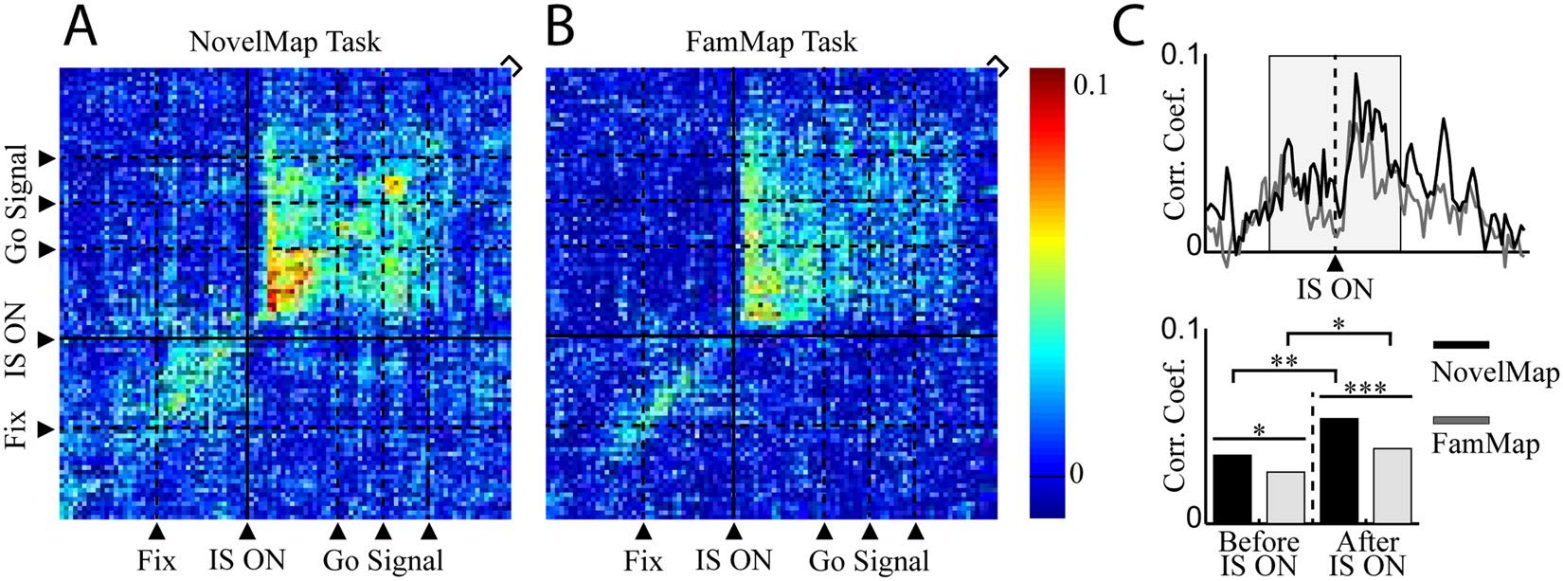
Population analysis aligned on the IS onset. (**A**,**B**) Average JPETHs of the neuron pairs significantly positively correlated in at least one task in the IS epoch. The average maps include 55 neuron pairs. As in Fig. 3, the bracket on the upper right corner represents the main diagonal. (**C**) Upper panel. Average coincidence histograms representing the correlation coefficients of the main diagonal of the average JPETHs for the studied epoch. Bottom panel. Average of the correlation coefficient before and after the onset of the IS in both tasks (between tasks, paired t-test; same task between epochs, Wilcoxon test, ^*^p < 0.05, ^**^p < 0.01, ^***^p < 0.001).

After showing that more pairs showed a positive significant correlation in the NovelMap task in the IS epoch we examined the correlation between neuron pairs at the population level. We computed a population average of the coincidence histograms of the significant pairs in at least one of the two tasks to analyze the time course of the correlation and the power of the correlation at the population level. Figure 4 shows the average JPETH of the significant neurons in at least one task during the IS epoch (n = 55) in the NovelMap (Fig. 4A) and the FamMap tasks (Fig. 4B) and the coincidence histograms of these average JPETH (Fig. 4C). We observed two main results with the analysis of these cross-correlated activity before and after the appearance of the IS in both tasks. First, the average correlation coefficients were significantly higher after, rather than before, the IS onset for both tasks (Wilcoxon rank sum test, V < 117, p < 0.05), indicating that in both tasks the increase in cross-correlated activity followed the IS onset. Second, the average correlation coefficient was higher in the NovelMap task than in the FamMap task both before (paired t-test, t = 2.46, p < 0.05) and after (paired t-test, t = 4.25, p < 0.001) IS onset. Therefore, the population analysis and the temporal profile of the cross-correlated activity revealed that the average correlation coefficient was higher in the NovelMap than in the FamMap task, not only after, but also before, IS onset. This difference was enhanced by the IS presentation.

## Discussion

The aim of our study was to test whether the learning of novel stimulus-response (S-R) mappings involved changes in the trial-by-trial spike-count correlation of the neuronal activity in the PFC in comparison to the retrieval of well learned associations. For this purpose, we compared the number of significantly correlated pairs of neurons recorded during two tasks, one in which the S-R mapping was already familiar and the other in which learning was required. We showed that the learning of the S-R mapping involved higher synchronized activity in a larger population of PF pairs of neurons during stimulus presentation. We also observed a tendency of a higher correlated activity across all the other task epochs.

Others have shown that the magnitude of neuronal correlations during execution of behavioral tasks can vary dynamically during cognitive operations^45,46^ and that patterns of firing correlation between single cortical neurons in behaving monkeys can be modified in relation to sensory stimuli and behavioral events, even in the absence of neuronal firing rate modulation^5^. The functional connection between two neurons can be potentiated or depressed based on the behavioral context^47^. Spiking activity synchronization is an established neural correlate of working memory and decision making processes and it may be responsible for performance improvement during working memory tasks^48,49^. Our study revealed an increase in the number of significantly correlated pairs and their correlation strength after stimulus presentations during learning in the NovelMap task compared to the FamMap task. Consistent with previous studies, our results showed that cognitive operations, in our case the learning of new S-R associations, are associated with concomitant higher spike-count correlations.

Qi and Constantidinis^10^ showed that learning and performance of a cognitive task alters the correlation structure in the PF. They compared the spike-count correlations of different population of pairs of PF neurons before and after the training of a working memory task and observed that the overall spike-count correlation declined after training. This correlation decrease occurred even when controlled for a potential confound represented by differenced in firing rate and was more pronounced during the two stimuli presentation. A similar decrease of spike-count correlation was observed for an attended versus an unattended stimulus by Cohen and Maunsell^50,51^. In our study, we did not directly compare the spike-count correlations of all recorded pairs but we restricted the analyses to the number of pairs showing a significant correlation during the stimulus presentation. Nevertheless, our results showed less correlated pairs during the stimulus presentation for familiar S-R mappings. In line with the previous studies, this number of correlated neuron pairs could reflect the monkey’s familiarity with the stimuli. The knowledge of the meaning of the stimuli in the context of the S-R mappings could be represented by a reduction of the neuron pairs with correlated activity and, in contrast, the learning process might be associated with more pairs with correlated activity.

The relationship between correlations and the amount of information in a population code is a current topic of interest. However, the most widespread hypothesis suggests that correlated neuronal responses decrease information encoded by a population of neurons^52,53^. According to this theory, the covariation in firing rate could reduce the signal reliability obtained by pooling different information by the downstream neurons^54^. Indeed, the covariation in firing rate and the sharing of common noise can limit the fidelity of signal transmission^55^. In their studies, Constantinis and colleagues^10,56–58^ observed that the training of an animal and the improved representation of stimuli in PFC neurons was obtained through mechanisms independent from single neuron firing rate and that it was mainly the correlation structure of neuronal firing which explained the improved representation. Our results extend the knowledge on the functional connectivity of PFC to the learning of novel associations in the context of a well-defined task structure. Indeed, as in the study of Qi and Constantidinis^10^, in which the decrease in neuronal correlation has been interpreted as been useful to improve and refine the stimulus representation, we can hypothesize that in our experiment a lower number of correlated pairs with lower correlation in the FamMap task could refine the S-R associations previously learned. On the other hand, the larger number of correlated pairs found in the NovelMap task might reflect the homogeneity of the neuronal activity when facing the learning of novel mappings and limit the representation of the novel stimulus by downstream neurons.

The link between functional connectivity and processes such as learning and memory is a key component to be investigated to understand how neurons preserve stable changes across time. Baeg *et al*.^59^ showed that in behaving rats the correlated firing of PFC neurons changed significantly during the early phase of a training and that the altered functional connectivity remained constant over time when the learning reached an asymptote. Our results revealed that fewer neural pairs of PFC showed a significantly correlated firing rate when the stimuli were already well associated with a corresponding behavior leading to the outcome. The need to process a large number of trials for each stimulus did not allow us to separate the recording session into separate phases. Thus, we were unable to study the time course of learning in the NovelMap task. Our results suggest that the number of correlated pairs might decrease during learning. This hypothesis is consistent with the cell assemblies’ theory of Hebb^1^ which postulates that the patterns of connectivity in a group of frequently coactivated neurons is altered by learning^1^.

## Material and Methods

### Subjects and behavioral task

Two adult male rhesus monkeys (Macaca mulatta), 8.8 kg and 7.7 kg, were studied. Each monkey sat in a primate chair, with its head stabilized and faced a video monitor 32 cm away. All procedures conformed with the guide for the care and use of laboratory animals (1996, ISBN 0-309-05377-3) and were approved by the appropriate animal care and use committee. A fluid control was used to motivate the monkeys to perform the task.

Figure 1A–C illustrate the tasks, first used by Genovesio *et al*.^19^. In both tasks, the mapping task (NovelMap task) and the familiar mapping task (FamMap task), a trial began after 2.5 s intertrial interval, when a white circle (0.7° visual angle), called the fixation spot, appeared at the center of the video screen. Once the monkeys fixated this location, three potential saccade targets (2.2° unfilled white squares) appeared 14° from the fixation point. After the monkeys had maintained fixation on the fixation spot (±7.5°) for 1.0 s, the fixation spot disappeared and a symbolic visual instruction stimulus (IS) appeared in place of the fixation spot for a pseudorandomly selected period of 1.0, 1.5 or 2.0 s. Each IS was composed of two colored ASCII characters superimposed. The offset of the IS also served as a “go” signal, after which the monkeys had to make a saccade to one of the three potentials goals (±7.5°). The monkeys had to fixate the chosen goal for 1.0 s, then all 3 response targets filled in white and a 0.1 ml drop of fluid reward occurred 0.5 s later if the response matched the mapping rule. Regardless of rewarded or unrewarded trials, the response targets disappeared from the screen at that time and a 2.5 s intertrial interval began. During the FamMap task (Fig. 1B), the monkeys had to respond to highly familiar ISs. Those ISs were already known according to three well-learned stimulus-response mappings that monkeys encountered during the strategy task (see Genovesio *et al*.^19^). During the NovelMap task (Fig. 1C), the monkeys learned three novel mappings of the same nature as in the FamMap task. The difference between the two tasks lies in the fact that all three mappings were unknown at the start of a NovelMap task. A correct response was followed by the delivery of reward and an error by an absence of reward as a feedback. After each incorrect choice, the same IS was presented again until the monkey chose correctly.

### Surgery

A 27 × 36 mm recording chamber was implanted over the exposed dura mater of the right frontal lobe using aseptic techniques and isoflurane anesthesia (1–3%). We implanted titanium bone screws in the surrounding bone and used methacrylate cement to affix the chamber and the head restraint device to these screws. After the operation, analgesia was given for 3–5 days.

### Histological Analysis

Near the end of data collection, we made electrolytic lesions (15 µA for 10 s, anodal current) at two depths per penetration. After approximatively 10 days, the animal was deeply anesthetized, 5 localization pins were inserted at known chamber coordinates, and then the animal was perfused with buffered formaldehyde (3% by weight). The brain was cut coronally in 40 µm sections on a freezing microtome, then Nissl stained. The surface projections of the recording sites were plotted by reference to the electrolytic lesions and the pins. Figure 1D shows the location of the recordings in the dorsolateral prefrontal cortex (dlPFC) and the dorsomedial prefrontal cortex (dmPFC).

### Recording methods

The position of each monkey’s gaze was recorded with an infrared oculometer (Bouis Instrument, Karlsruhe, Germany). Single-unit potentials were isolated using a 16-electrode microdrive with independent control of each electrode (Thomas recording, Giessen, Germany) through a custom, concentric recording head with 518 µm electrode spacing. The signal from each quartz-insulated platinum-iridium electrode (impedance, 0.5–1.5 MΩ at 1 kHz) was amplified and discriminated using a Multispike Detector (Alpha-Omega Engineering, Nazareth, Israel) or a Multichannel Acquisition Processor (Plexon, Dallas, TX). For the latter, we resorted neuronal waveforms with Offline Sorter (Plexon). NIMH CORTEX software (https://www.nimh.nih.gov/labs-at-nimh/research-areas/clinics-and-labs/ln/shn/software-projects.shtml) controlled behavior and collect data.

### Data Analysis

We used the Matlab (http://www.mathworks.com) and the Fieldtrip Matlab toolbox^60^ (http://www.ru.nl/neuroimaging/fieldtrip) to analyze the data. For subsequent analysis, we selected neurons that were recorded in both tasks for at least 50 trials in each task. We applied the Joint Peri Event Time Histogram (JPETH) analysis to the pairs of simultaneously recorded neurons (Aertsen *et al*.^36^). The JPETH analysis was performed on each selected neuron pairs by building the two-dimensional cross-correlograms with time versus lag on both abscissa and ordinate axis and the correlation strength on the color axis. The activity from a neuron *i* is represented as ${S}_{i}^{r}(t)$ for the *r*th trial^61^. The averaging over r trials is represented by 〈〉 and *P*_*i*_(*t*) is defined as ${S}_{i}^{r}(t)\,\,$and is the averaged response or PETH of a neuron *i*. For each pair, we calculated the raw JPETH and, also, the shuffle predictor. This predictor was calculated by averaging the JPETH across 1000 possible permutations of the trials. We then computed shuffle-corrected covariance matrices, known as covariogram which were defined as:

$${J}_{i,j}({t}_{1},{t}_{2})=\langle {S}_{i}^{r}({t}_{1}){S}_{j}^{r}({t}_{2})\rangle -{P}_{i}({t}_{1}){P}_{j}({t}_{2})$$

To compute the covariogram, the shuffle predictor was substracted from the raw JPETH to correct for correlations originating from covariations originating from direct stimulus effects on firing rates^35^. Next, to normalize the covariogram and obtain a matrix of correlations coefficients bounded between −1 and 1, the above equation was divided by the cross product of the time-dependent standard deviation (SDs) of the neurons i and j as follows:

$${J}_{Ni,j}({t}_{1},{t}_{2})=\frac{{J}_{i,j}({t}_{1},{t}_{2})}{{\sigma }_{i}({t}_{1}){\sigma }_{j}({t}_{2})}$$

Most of the temporal range of correlation is encompassed in a strip of bin near the main diagonal^5^. Consequently, we plotted the correlations in these bins using the average of the main diagonal and the two surrounding diagonals to have the equivalent of 150 ms along the main diagonal with 50 ms window bins^38^. These histograms of the main diagonal coincidence were then used to select the significant neuron pairs based on statistical analysis described below. For each neuron pair, the correlation coefficient of each 150 ms bin of the main diagonal was transformed into *z* value using the Fisher’s transformation:

$${z}^{1}=\frac{1}{2}ln\frac{(1+r)}{(1-r)}$$

where *z*^1^ has a standard deviation of $S{D}_{{z}^{1}}=\frac{1}{\sqrt{n-3}}$ and where n is the number of trials. From these values we defined 95% confidence interval as $CI=tanh({z}^{1}\pm {z}_{\alpha /2}S{D}_{{z}^{1}})$ to perform our statistical analyses. Because this analysis was performed on 40 bins during each analyzed epoch described below, we divided the significance level with Bonferroni correction at 0.025/40 = 0.000625. The corresponding value of the corrected 95% confidence interval limits ${z}_{\alpha /2}$ (initially 1.96) was then 3.23. We performed this analysis during 4 epochs, from 1 s before to 1 s after, the “IS”, the “go signal” (disappearance of the IS), the “target fixation” and the “reward”. The reward occured in the correct trials and the absence of reward provided error feedback. All correct and error trials were included in the analysis. Some artifacts could arise from covariations in the neuronal excitability or variability in task events and such artifacts are sensitive to bin size^61^. Consequently, we performed the same analysis on the significant-correlated neuron pair using small-size bins of 20 ms (Supplementary Fig. 2A–F, G–L shows the example of the neuron pair presented in the Fig. 3 using these 2.5 time smaller bins).

### Data availability

The datasets generated and/or analyzed during the current study are available from the corresponding author on reasonable request.

## Electronic supplementary material

Supplementary information
