# Supplementary material for: Learning the meaning of new stimuli increases the cross-correlated activity of prefrontal neurons
Source: Sci Rep. 2018 Aug 3;8:11680. doi: 10.1038/s41598-018-29862-0 (PMC6076274; doi:10.1038/s41598-018-29862-0)
Supplement: Supplementary file 1 — Supplementary information [file 41598_2018_29862_MOESM1_ESM.pdf]

Title

Learning the meaning of new stimuli increases the cross-correlated activity of prefrontal neurons

Simon Nougaret[1] and Aldo Genovesio[1]*

[1]Department of Physiology and Pharmacology, Sapienza University of Rome, Piazzale Aldo Moro 5, 00185 Rome, Italy.

*Correspondence to: aldo.genovesio@uniroma1.it

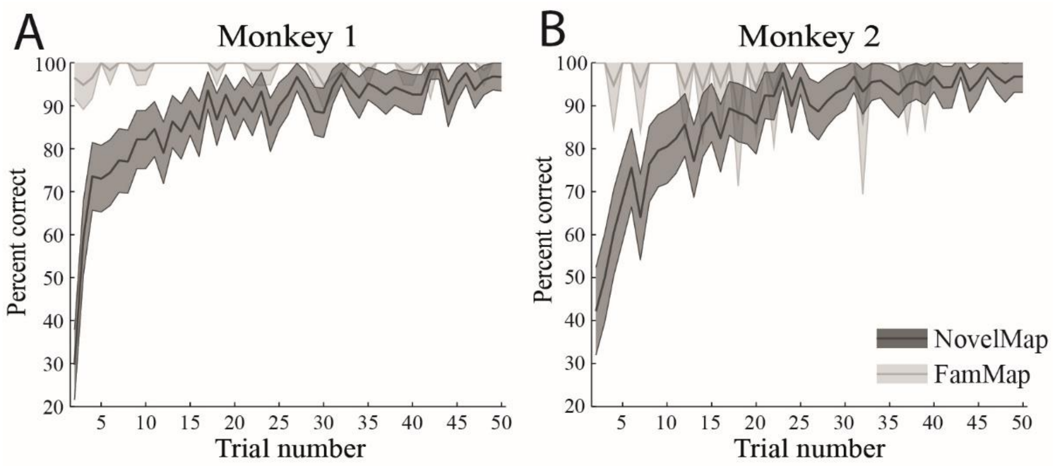

Sup. Figure 1

Behavioral performance computed separately for each monkey. A. Percentage of correct responses in the first 50 trials for each task averaging for Monkey 1. The light gray curve indicates the performance during the FamMap task and the dark gray ones the performances in the NovelMap task. B. Same as A for Monkey 2. Background shading indicates 95% confidence limits.

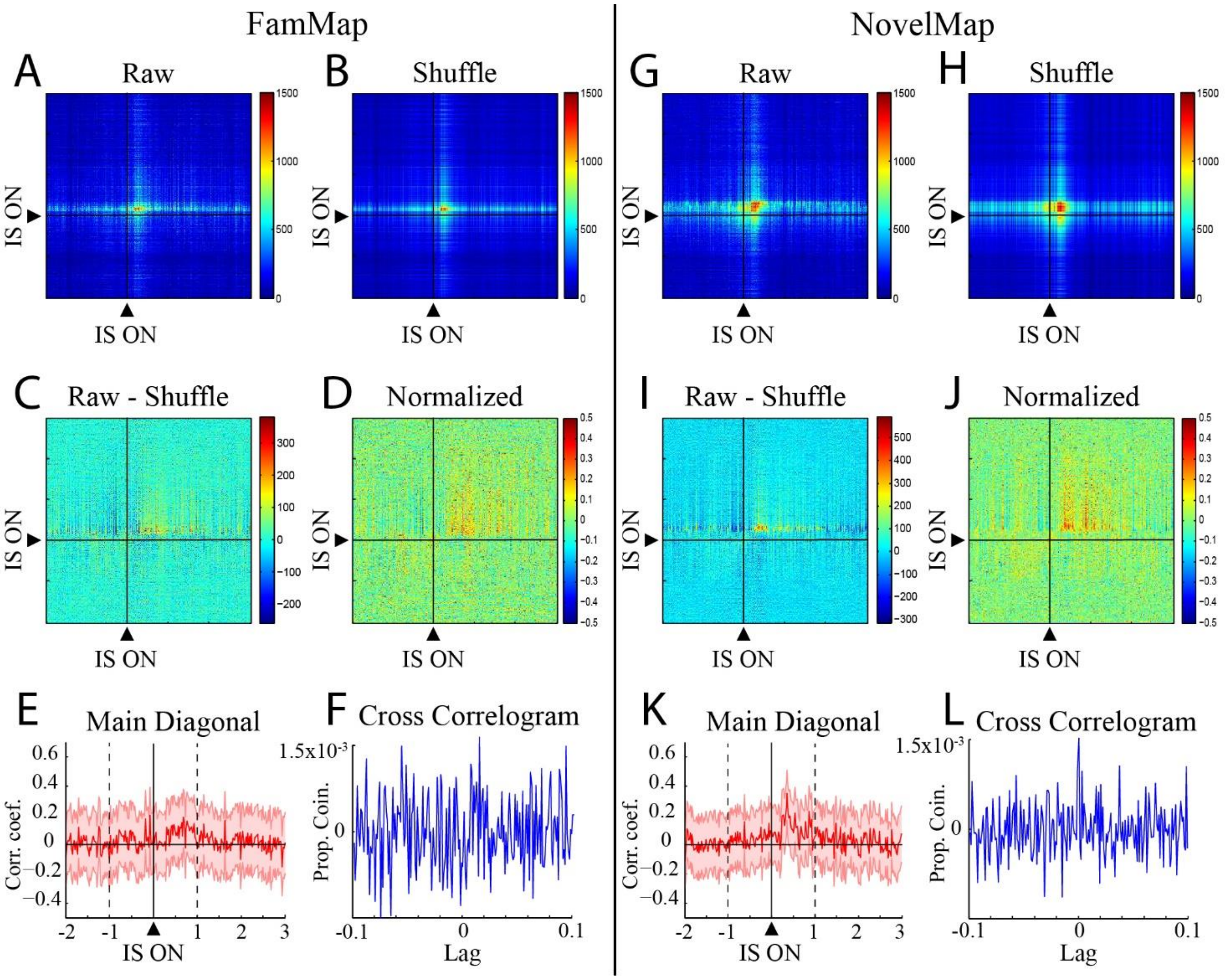

Sup. Figure 2

JPETH and cross-correlogram of the same neuron pair presented in Figure 3 with different bin size. The left panel displays the results for the FamMap task and the right panel for the NovelMap task. A, G. Raw JPETH with bin size of 20 ms aligned on the IS onset. B, H. Shuffle predictor during the same period with same parameters. C, I. Shuffle JPETH subtracted from the raw JPETH. D, J. Normalized JPETH (Raw JPETH – Shuffle JPETH divided by the cross product of the time-dependent standard deviation) highlighting the trial-by-trial correlation of this neuron pair after IS onset. E, K. Coincidence histogram representing the correlation coefficient along the main diagonal with the uncorrected 95% interval. F, L. Corrected cross-correlogram with time bins of 1 ms and maximal time lag of 100 ms.

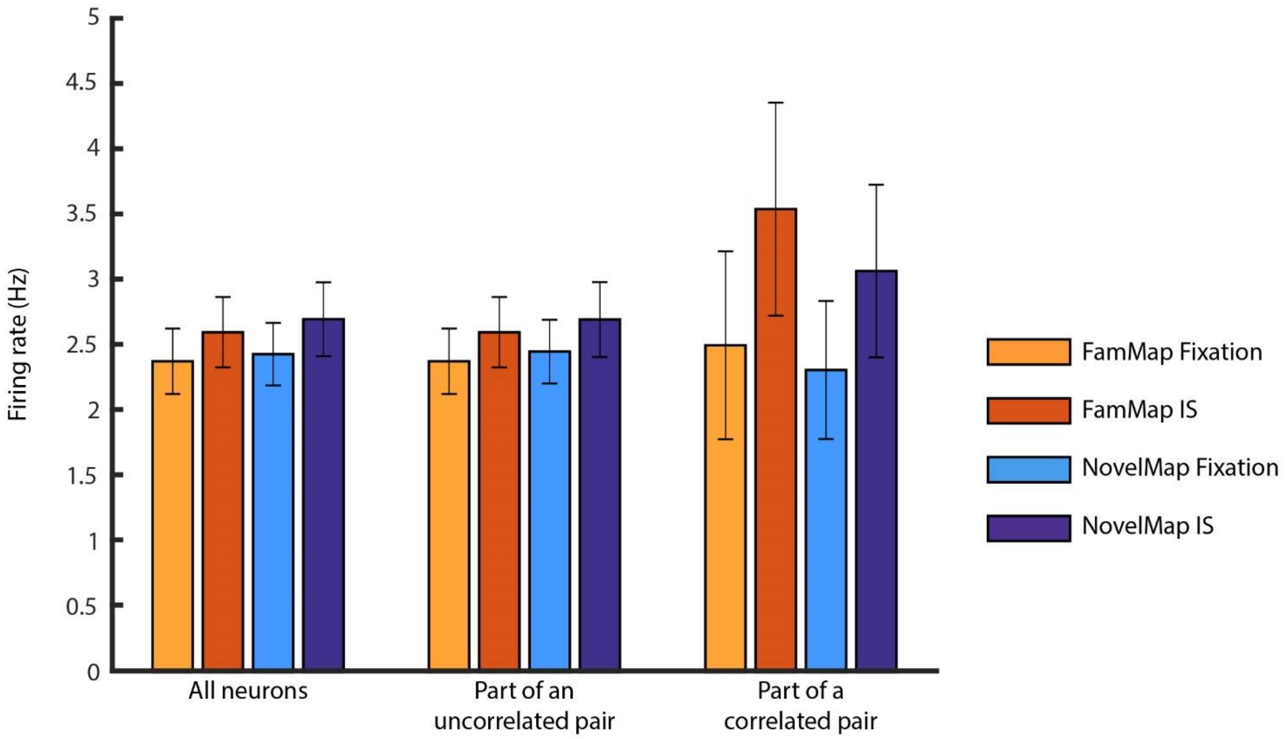

Sup. Figure 3

Firing rate of the neurons in both tasks during the fixation period and the first second of the IS. The four bars on the left show the firing rate of all the 224 neurons making up the 439 analyzed pairs. The four bars on the middle show the firing rates of neurons which are part of an uncorrelated pair (224 neurons in the FamMap task and 220 in the NovelMap task) and the four bars on the right show the firing rates of neurons which are part of a correlated pair (34 neurons in the FamMap task and 65 neurons in the NovelMap task). Most of the neurons were part of both the correlated and uncorrelated pairs. We found no significant difference in firing rate between the FamMap and the NovelMap tasks. There was also no significant difference in firing rate between correlated and uncorrelated pairs in both the FamMap and the NovelMap tasks.
